# A Cross-Level Investigation of Team-Member Exchange on Team and Individual Job Crafting with the Moderating Effect of Regulatory Focus

**DOI:** 10.3390/ijerph17062044

**Published:** 2020-03-19

**Authors:** Tsang-Kai Hung, Chih-Hung Wang, Mu Tian, Ya-Jiun Yang

**Affiliations:** 1 National Changhua University of Education, Changhua 50007, Taiwan; 2 East China University of Science and Technology, Shanghai 200237, China

**Keywords:** team-member exchange, team job crafting, individual job crafting, regulatory focus

## Abstract

Within the framework of regulatory focus theory, this study examines the issues of job crafting. This study adopts purposive sampling as a means to collect data. A total of 123 teams with 514 members were invited to participate in the survey, and 91 teams with 354 members provided valid questionnaire responses for data analysis. Mplus 7.0 was applied to conduct data analysis and verification. Data analysis demonstrates that (1) team-member exchange (TMX) exerts a positive influence on team job crafting and individual job crafting; (2) team job crafting positively affects individual job crafting; (3) TMX can positively affect individual job crafting via team job crafting; and (4) a prevention focus has a moderated mediation effect on the indirect relationship between TMX and individual job crafting. Based on its findings, this study has both practical and theoretical implications. Academically, it can be regarded as a pioneering academic endeavor. Practically, this study can enhance teamwork, postulate job flow, and promote the quality of member relationships, thus boosting individual job crafting performance.

## 1. Introduction

In recent years, organizational studies have placed increased emphasis on job crafting. Job crafting can positively influence individual employees and teams, including enhancing employee job satisfaction, commitment, and performance, and reducing turnover rate [1,2,3,4]. Job crafting not only benefits individual job performance but also satisfies organizational expectations of job behavior. However, past studies have focused on predictions of employee job performance but have neglected the causes and means of motivating individual job crafting. Therefore, this area needs more focus and academic endeavor. It is one of the motivations of this study to understand the causes accounting for employees’ job crafting.

Job crafting studies have tended to be concerned with employees. Recently, however, more emphasis has been placed on team job crafting [2,5,6]. A team is, practically, a basic unit meant for completing a job [7]. For this reason, in [8], it was regarded as necessary to study team job crafting. Related studies of team job crafting show that it can positively affect team job satisfaction, commitment [5], and job engagement and performance [2,6]. Nevertheless, little knowledge exists about the causes of team job crafting. In [3], the importance of both interpersonal relationships between team members and personal influence was emphasized. Therefore, cross-level influence deserves more attention [9]. An understanding of the causes of team job crafting is thus the second motivation of this study.

Employees’ organizational behavior is subject to environmental influences; conversely, team members’ influence is the most direct [10]. A recent study of social community, teamwork, and social resources has suggested that interpersonal relationships among coworkers can lead to meaningful results [11], such as knowledge sharing and organizational civil behavior [12]. The theoretical model of job crafting, according to past studies, needs to take social factors in a work environment into consideration, particularly amongst coworkers and teamwork [13]. In view of this, this research investigated whether the quality of member relationships influences the team and individual job crafting by referring to member relationships as the preceding variable of the relationship between team and individual job crafting.

In [14], it was argued that employees’ interests and personality traits account for their use of a social community. According to that study, personal initiative and a proactive personality are related to individual job crafting. Employees with those two personality traits can exhibit higher job crafting behavior [1,15]. However, this can only predict the degree of individual job crafting, and it does not explain whether differences in personality traits have a moderating effect on job crafting [16], and only distinguished a “promotion focus” from a “prevention focus.” Members with different inner needs and cognitive styles are likely to implement different strategies and following behaviors. This study investigates whether members with different regulatory focus traits interfere with the relationships of team-member exchange, the indirect relationship of individual job crafting, and whether different traits exert an influence on personal behavior.

This study makes three contributions. First, by investigating the influence of team-member exchange relations on team job crafting and individual job crafting, it fully explains the influence of human relations and social connection on job crafting. Therefore, it fills an academic area little dealt with before. Second, it investigates the mediating effect of team job crafting, that is, whether the quality of team interaction can affect individual job crafting through team job crafting. Third, it further investigates how the personal regulatory focus trait interferes with the above-mentioned relationships, that is, whether the personal personality trait enhances or reduces the influence of the team scenario on personal behavior.

## 2. Conceptual Boundaries

Before we elaborate our research method and process, it is necessary for us to clarify the concept of culture and innovation for our research objectives.

### 2.1. Job Crafting

Job crafting is defined as individuals shaping their work style and interacting with others through expanded or reduced tasks, relationships, and cognitive boundaries [8]. Then, they reshape the work of design and the work environment, reconstructing personal work meaning and job identity. In [17], the authors stated that job crafting is employee self-conduct behavior that incorporates personal motivation, strengths, and enthusiasm in the job, thus making the job more in line with the individual’s ability. They suggest that employees can change their job roles and work by changing three job practices: (1) task boundaries: people can change the type and number of individual tasks, and determine how to complete their task in a different way; (2) relationship boundaries: people change interactive objects and the essence of interaction; i.e., individuals can decide who interacts and the quality and frequency of interactions; and (3) cognitive boundaries: people change the individual’s view of the work, i.e., the way a person looks at his work.

To be able to more accurately capture actual job crafting behavior, employees can shape their job by changing their work characteristics; in [15], job crafting was defined as when “employee autonomy changes their work so that individuals can optimize personal work needs and work resources to achieve work goals.” Furthermore, in that work, job crafting was divided into two categories and four sub-concepts in detail based on the theoretical model of job demand resources: (1) increasing structural job resources: increasing work diversity and developing opportunities and autonomy to improve working resources; (2) increasing social job resources: increasing job support, supervisory guidance, and feedback to improve work resources; (3) increasing challenging job demands: increasing challenging job requirements to improve employee work skills and knowledge development; and (4) decreasing hindering job demands: reducing non-essential job requirements that will affect personal goals achieved and exceed an individual’s ability.

### 2.2. Team-Member Exchange (TMX)

In [18], the author, based on role theory and social exchange theory, proposed the concept of team-member exchange (TMX), which is the process of reciprocal exchange between team members, including a member offering help, ideas, and feedback for others, and the degree of obtaining information, help, and recognition from other members. TMX reflects the individual’s overall perception of the quality of the work relationship within the team. The growth of TMX is based on interacting between each other; this will affect team member’s behaviors and attitude [19]. There is full interaction and expression of thoughts with a high quality of TMX. Members understand other members’ potential, ability, and work value and job identity [20]. In order to keep a positive self-image and better fit the goal and work role that the team expects, an individual will change self-job perspectives.

### 2.3. Regulatory Focus

In [21], it was pointed out that the motivations of job crafting will meet the needs of job satisfaction and change the work of action and work meaning through reaching goals. Hence, motivations play an important role in strengthening job crafting when people are integrated with the motivation, achievement, and goals. Therefore, we select the regular focus as our moderated variable. In [16], the authors stated that, when individuals are achieving their goals, they will adopt two different self-regulating systems, namely, a “promotion focus” and a “prevention focus.” There are many similarities between the two concepts; for example, both concepts are divided into positive and negative, and the basic behavior and motivation involve efforts to achieve the goal through different strategies. Hence, we think the regular focus can present different motivations for individual job crafting behavior.

## 3. Research Hypotheses

### 3.1. Relationship between Team-Member Exchange and Individual Job Crafting

According to social cognitive theory, the processing of human beings is based on mutual influences of the individual, behavior, and environment. In [22], it was reported that personal cognition plays a key role in fact construction, self-regulation, coding information, and behavior manifestation. As a result, during team-member interactions, some members’ verbal and behavioral transmissions of their perspectives about a specific event/incident may change other team members’ behavior and attitudes [18]. The interactions between members can help them construct job content, such as job characteristics, job type, and job-performing methods. All these not only help define the job model and postulate a mission’s domain [8], but they also further create job identity. In addition, high-quality team-member exchange relationships allow members to fully interact with each other and freely express their thoughts. Members can be evaluated by other members as to their ability, job value, and job role.

Interpersonal interactive behavior, in terms of resources, is a process in which participants are engaged in an activity relevant to them. They possess and exchange valuable resources [23]. Supervisors and coworkers may provide useful feedback and resources, and their discussions about job competition are very likely to affect other workers’ learning and innovation. It can also make a mission more challenging and diversified, thus elevating the degree of job crafting [5]. According to this study, team members not only help other members understand their job role and mission content, but they can also transmit their perspectives about the job and provide sufficient resources by means of communication and interflow.

**Hypothesis** **1.** *Team-member exchange relationships can positively affect individual job crafting.*

### 3.2. Team-Member Exchange Relationships and Team Job Crafting Relationships

On-the-job interpersonal relationships, which extend and develop with social interactions and mission interaction time, are the basis of group activity [24]. According to the concept of group activity in social cognitive theory, when a group shares collective power (force), they can turn out a common belief and possess the same mission objective. Team members have a group-identified direction and convert personal interest into group interest, and work together for an expected goal [25]. In addition, in order to accomplish the same mission, members not only need commonly shared knowledge and skill, but also must communicate and work together through interaction and effective communication to generate effective outputs [26]. Individuals tend to work with those better associated with themselves [11]. With the quality of member interactions becoming higher, team members can freely express their own thoughts and share others’ knowledge and skill, thus enabling them to have common beliefs about collective efficacy and expected outcomes. They define job flow, jointly create job resources and job requirements, and enhance team job crafting to achieve common objectives [2].

According to [5], social connections and interactive relationships between teachers and their assistants can positively affect cooperative job crafting, change the job environment and the job performing method, and uplift care quality. Therefore, this study suggests that good relational quality can provide team members with more knowledge sharing and feedback. With more resources and confidence, they can together determine an appropriate job method. They can, through effective communication and interflow, create a more suitable job environment for the team, share job resources, meet job needs, and realize a common objective.

**Hypothesis** **2.** *Team-member exchange relationships can positively affect team job crafting.*

### 3.3. The Relationship between Team Job Crafting and Individual Job Crafting

Team job members can influence one another. Some team members’ emotions and behaviors can affect others’ personal emotions and behaviors [27]. According to group rules and social cognition theory, group rules define personal behavior, and the individuals display actual behavior by imitating others. The group rule dictates members’ common beliefs about expected behavior, which can guide personal behavior [28,29]. If a team can build up a common mission, rule, behavioral mode, and interaction relationship in a short time, members can have something to refer to or rely on [30]. Group rule is quite influential. Conforming to a group rule can likely cause members to feel stressed to a certain degree [31]. Therefore, when a group rule is applied to the group, it is sure to affect the individual members. If members solve a group problem proactively, team job crafting will then send out a signal to urge the individual members to actively change job characteristics.

Individual members in these groups develop knowledge of the group rule through observing other members’ behaviors and responses [32] and are more likely to participate in other members’ job crafting. Members can judge which behavior is fitting for a certain work scenario. They must take into consideration job characteristics, including similarity, status, and success, so they can judge whether the behavior is worth imitating and whether it leads to a valuable result.

**Hypothesis** **3.** *Team job crafting can positively affect individual job crafting.*

### 3.4. The Mediating Effect of Team Job crafting

Social relations provide members’ actions with direction and create safe environments through knowledge sharing and group members’ changes [33,34]. As a result, the higher the quality, the higher team members’ interactions become, and the greater the inspiration for knowledge sharing and resource interflow. Members accomplish a common objective by defining the job objective and job flow, collectively creating job resources, satisfying job needs, and strengthening job crafting among the members [2].

In addition, the members’ interactive process can exhibit a collective sense of efficacy [35]. With the provision of new resources and changing of job environments, employees can engage themselves in the social learning process by following group beliefs and common beliefs [28]. Members do not have to experience errors. However, they can learn new behavior through observing others’ behavior [35]. Once team members share the job experience and complete a mission, they can create a propaganda effect. Coworkers help them improve the job environment and add to their personal environmental adjustment [13,15]. Team members can motivate others to complete their personal mission. For example, when team members believe they alone can carry out and complete a mission, individual members cannot only imitate them but become more confident in performing their own role. Team members’ self-expectations can be obtained through providing more social resources or making a job mission more challenging [2]. According to [2], job modelers share with each other what they gain from the job, learn skills acquired by others, improve their own skills and interpersonal relations, and redefine their own job identity.

This study suggests that high-quality relationships help members share knowledge and resources with each other, and jointly change their job environments. They jointly define job flows, job objectives, and job methods. They obtain more job and relationship resources to balance job needs and complete the mission. The individuals observe and learn such value-based behavior as exhibited by other members [22]. This helps them define their role, develop effective beliefs, and change their perspectives about their job and job method [5].

**Hypothesis** **4.** *Team-member exchange relationships positively affect individual job crafting and can regulate a moderated mediating effect through team modeling behavior.*

In [36], the combined effect of moderated mediation and mediated moderation is discussed. Moderated mediation means that any path (direct effect, indirect effect, or combination of both) can be influenced by another moderation variable [37,38]. In [5], it is suggested that larger job environments and job characteristics can motivate employees’ job behaviors. An employee’s conception of the job environment, job characteristics (personality trait, for instance), and personal preference for and orientation toward a job (motivation for achievement, for instance) affect the employee’s job crafting occurrence rate and intensity. Additionally, an employee can simultaneously take part in personal and cooperative job crafting. This means there are still other influential moderation causes pertaining to a job environment.

In [8], it is pointed out, on the basis of the job crafting model, that the motivation for job crafting can satisfy the needs of job achievement. It can change job action and meaning by enhancing one’s sense of the job objective. In addition, a close combination of personal factors and job objectives will strengthen each other’s roles. A past study has also suggested that employees with personal initiative and a proactive personality can display higher job crafting [15,39]. This implies that personality traits and motivation can interfere with an environment regarding its influence on personal behavior.

According to [16], people with different personality traits may adopt different strategies owing to different needs. People with a promotion focus care more about whether there is an opportunity for growth and progress. They search for an expected objective, and they also actively adopt a less distant objective strategy for a good result. People with a prevention focus, on the contrary, place more emphasis on personal security and assurance, and are very sensitive to the loss of resources. When searching for an objective, they tend to rely on personal self-conscious obligation, duty, and responsibility. They work with deliberation and caution, and try their best to avoid making mistakes [40,41,42]. In other words, someone’s personal cognition ability dictates their behavior. It makes an individual employee understand what objective to achieve and the amount of effort to spend [22]. Past empirical research indicates that people with a different regulatory focus respond and behave differently to the same event/incidence, such as group-decision behavior, as a result of different cognitive styles [43].

It can be expected, therefore, that people with a different regulatory focus will also adopt different behaviors and strategies when faced with environmental influences. Two different strategies are meant to either allow individuals to access the expected status (promotion focus) or help individuals avoid an expected dilemma (prevention focus) [41]. Hypothesis 4 proposes that the quality of team relationships can affect personal emotions and behaviors. Through in-group interactions, team members can offer new job resources and change the job environment by following the team’s collective beliefs and experiences [28]. Social relationships can present individuals with a direction to follow. When performing a job, employees can imitate and follow other members’ outwardly valuable behaviors [22]. Subsequently, they can judge the appropriateness of behaviors by observing what others do. Therefore, those who fall into the prevention focus category follow the situational clues manifested by social relationships for the sake of their security needs. When performing a job, they tend to exhibit safe behaviors that can be reproduced or imitated. When they make a decision, they are concerned with the team’s objectives and interests, and are sensitive to negative outcomes and loss of resources. In [44], it is suggested that people with a prevention focus are likely to feel stressed when making a suggestion; thus, they will eventually become psychologically exhausted. They prefer to maintain the status quo to prevent unwanted events/incidents from occurring. They are careful about decision making to avoid making mistakes [45]. They follow the team rule and adjust personal behavior by referring to the overall job crafting. In contrast, those with a promotion focus are more willing to take and assume risks to enhance opportunities and thus complete their objectives [45]. Consequently, the situational clues and resources provided by social relationships become merely one way to access an objective but not the only way to accomplish it. If in-group interaction quality and team job crafting frequencies are low, the team’s scenario may not necessarily influence these employees. Instead, they use their own way to search for personal growth and achievement by actively approaching the objective [45].

**Hypothesis** **5.** *Regulatory focus can moderate the indirect relationship between the team-member exchange relationships and individual job crafting. Those with a prevention focus can enhance the indirect relationship between team-member exchange relationships and individual job crafting. Those with a high-promotion focus are less likely to display a moderating effect.*

## 4. Methodology

### 4.1. Sampling and Procedure

This study originated from a project of MOST (Ministry of Science and Technology, Taiwan) regarding job crafting. The project was sent to the Ethics Committee of NCUE (National ChangHua University of Education, Taiwan) for sampling approval, and purposive sampling was employed to recruit participants. We selected from the population of industrial companies in Taiwan a stratified, proportional random sample of nearly 500 industrial companies. There were two activity categories, namely, industrial product and service companies. Participants gave informed consent. The questionnaires were distributed to 123 teams, and each team comprised at least three team members. Every team member had been in his or her position no less than three months. Every team member must have had a common goal, and similar job duties and work responsibilities. For teams with more than six members, more than half of the team members were required to return their inventories to be counted as a valid team. Finally, there were 108 teams, with 442 members returning their questionnaires. The response rate was 87.8%. Deleting invalid returned questionnaires resulted in 91 teams (or 364 valid questionnaires). The effective return rate was 84.2%. Among all participants, 65.1% were women; 26.1% participants were older than 41 years old, while 25.5% were aged between 26 and 30. Seventy-two percent (72%) of the participants had completed college or obtained a graduate degree. Of the total number of participants, 21.4% had one to three years of working experience, while 21.7% had over 10 years working experience. Regarding team size, most teams comprised 6–10 team members (41.5%).

**Figure 1:**
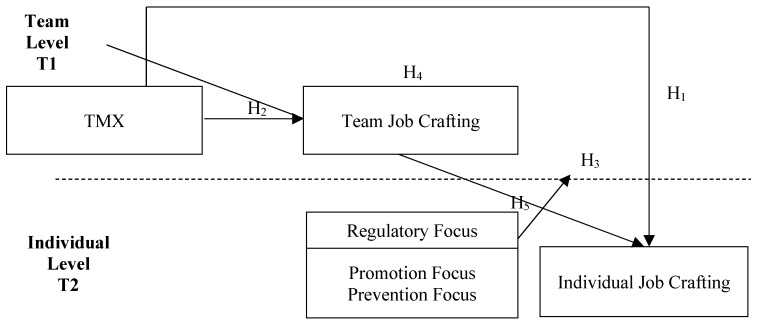
Research structure.

Data were collected in two waves. For the first wave, participants were requested to fill in “team-member exchange” and “team job crafting” questionnaires, and some demographic information that could be used as identification labels. The second wave questionnaires were distributed to the same participants one month after they returned their first wave questionnaires. The second wave questionnaires included “regulatory focus” and “individual job crafting,” and some demographic information that could be used to identify the participants. The returned questionnaires’ data for two waves were analyzed by the dyad method. Figure 1 shows the research framework.

### 4.2. Instruments

This study collected data from questionnaires covering team-member exchange, team job crafting, individual job crafting, regular focus, and demographics. We selected pilot testing to ensure the quality of questionnaires and measure the data we expected. According to the results of pilot testing, we revised or deleted questions to enhance the validity of the questionnaires. Lastly, we delivered the questionnaire to the companies. The content of measures and control variables was as follows:

*Team-member exchange*. Team-member exchange is defined by the relationship between members and colleagues in the group [18]. The team-member exchange questionnaires were adopted from part of the work of [18]. In the scale, numbers 1–4 measure the member’s perception of the team cohesion, numbers 5–14 measure the quality of members’ exchange relationship, and numbers 15–18 measure the effectiveness of team meetings. The Cronbach’s α measures of internal consistency were 0.80, 0.83, and 0.83, respectively. Because of the purpose of measuring the team-member exchange quality, we selected 10 items from numbers 5–14 as our measuring tool. We also used a Likert five-scale measure of team-member exchange, where 1–5 represented strongly agree, agree, neutral, disagree, and strongly disagree, respectively. A higher scale represented higher team-member exchange in this group. The measures for team-member exchange scale items are located in Table 1.

*Individual job crafting*. The 21-item individual job crafting scale [39] was used to measure individual job crafting. The measure was based on the development of the job demands resources (J-DR) theoretical model, which is divided into four facts, namely, increasing structural job resources, increasing social job resources, increasing challenging job demands, and decreasing hindering job demands. We selected 21 items to measure individual job crafting by employee self-assessment. We divided individual job crafting into two parts, namely, positive and negative, according to previous research [46]. The two parts of the individual job crafting scale were as follows.

Positive individual job crafting: This study was based on the development of the positive individual job crafting scale developed by [39], which includes three parts: increasing structural job resources, increasing social job resources, and increasing challenging job demands. The Cronbach’s α measure of overall internal consistency was 0.89 and had a total of 15 questions. Employees were asked to indicate the frequency of individual participation in job crafting. We used a Likert five-scale measure of positive individual job crafting, where 1–5 represented always, often, sometimes, occasionally, and never, respectively. A higher scale indicated that the employees showed more positive individual job crafting. The measures for positive individual job crafting items are located in Table 2.

Negative individual job crafting: This study was based on the development of the negative individual job crafting scale developed in [39], which includes only one part: decreasing hindering job demands. The Cronbach’s α measure of internal consistency was 0.72 and had a total of 6 questions. Employees were asked to indicate the frequency of individual participation in job crafting. We used a Likert five-scale measure of positive individual job crafting, where 1–5 represented always, often, sometimes, occasionally, and never, respectively. A higher scale indicated that the employees showed more negative individual job crafting. The measures for positive team job crafting items are located in Table 3.

*Team job crafting*. Team job crafting was the team level in our study, so we modified individual job crafting developed in [39]. Team job crafting was also divided into four facets: increasing structural job resources, increasing social job resources, increasing challenging job demands, and decreasing hindering job demands. We used the self-assessment measure for the level of team job crafting and then the average number of individual employees as the degree of team job crafting.

Positive team job crafting: This study was based on the development of the positive individual job crafting scale developed by [39] which included three parts: increasing structural job resources, increasing social job resources, and increasing challenging job demands, with a total of 15 questions. Employees were asked to indicate the frequency of team members showing positive job crafting behavior. We use a Likert five-point scale to measure positive team job crafting, where 1–5 represented always, often, sometimes, occasionally, and never, respectively. A higher scale indicated that each member of the team showed more positive job crafting. The measures for positive team job crafting items are located in Table 4.

Negative team job crafting: This study was based on the development of the positive individual job crafting scale developed by [39], which included one part: decreasing hindering job, with a total of 6 questions. Employees were asked to indicate the frequency of team members showing negative job crafting behavior. We used a Likert five-point scale to measure negative team job crafting, where 1–5 represented always, often, sometimes, occasionally, and never, respectively. A higher scale indicated that each member in the team showed more negative job crafting. The measures for negative team job crafting items are found in Table 5.

*Regulatory focus*. The study adopted the regular focus scale developed in [47]. They divided regulartory focus into two parts: promotion focus and prevention focus. Each part had 9 items to measure the concept, and the Cronbach’s α measures of internal consistency were 0.91 and 0.85, respectively. We use a Likert five-point scale to measure regulartory focus, where 1–5 represented as strongly agree, agree, ordinary, disagree, and strongly disagree, respectively. Higher scales for promotion than prevention indicated that subjects would be more aggressive toward goals. Higher scales for prevention than promotion indicated that subjects would adopt a conservative strategy to achieve goals. The measures’ items for promotion focus are found in Table 6 and for prevention focus in Table 7.

From the above results, the Cronbach’s α of all scales were above 0.70 (except team job crafting), which is in line with the acceptable standards in [48]. This indicates that the scale of this study had good reliability.

*Control variable*. In [49], work experience is examined. The authors found that it seems likely that, when individuals stay in one organization for a longer time, they develop knowledge about work processes, organizational arrangements, and culture. Moreover, experienced employees might have more knowledge about workflow and work processes, and more realistic expectations about which job crafting activities are therefore established. Furthermore, in [50], it is argued that some theorizing exists to support specific relationships between job crafting, and demographic and employment characteristics. It could be argued that employees with higher levels of education may have greater accumulated job and general knowledge and are thus in a better position to craft their jobs compared to employees with lower levels of education [50]. Therefore, educational backgrounds and work experiences were both control variables.

### 4.3. Aggregation of Group Level Data

This study examined Rwg, ICC(1), and ICC(2) (in statistics, the intraclass correlation, or the intraclass correlation coefficient (ICC), is a descriptive statistic that can be used when quantitative measurements are made on units that are organized into groups. It describes how strongly units in the same group resemble each other) for group level variable “team-member exchange” and “team job crafting.” The Rwg for team-member exchange ranged from 0.83 to 1.00 with a mean value of 0.92, while Rwg for team job crafting fell between 0.80 and 0.98 with a mean value of 0.90, which indicated that the Rwg value is reasonable. For between-group variance, the ICC(1) values between team-member exchange and team job crafting were 0.11 and 0.25; ICC(2) values were 0.34 and 0.56. ANOVA analysis showed that there were group differences between team-member exchange and team job crafting.

### 4.4. Overall Model Fit

The overall model fit indices were as follows: χ2/df was 2.40; GFI was 0.70 (the goodness of fit index (GFI) is a measure of fit between the hypothesized model and the observed covariance matrix); CFI was 0.92; NNFI was 0.87; RMSEA is 0.06. Other than the GFI value, the remaining values were acceptable. This indicated that the research model was still acceptable.

## 5. Results

### 5.1. Common Method Variance

To ensure the results did not suffer from the problem of common method variance (CMV), Harman’s one-factor test was employed to reveal that the variance accountable for the first factor was only 19.82%. Additionally, a cross-level study is a way to reduce common method variance; this approach is called unit isolation analysis [51].

### 5.2. Correlational Analysis among Study Variables

Table 8 shows that Pearson’s r indicated that there was a bivariate correlation between every pair of variables except for the pair of team-member exchange relationships and prevention focus. Moreover, the largest Pearson’s correlation coefficient was 0.56, which meant low collinearity threats among these variables.

### 5.3. Team-Member Exchange Relationships, Team Job Crafting and Individual Job Crafting

In order to realize the relationships among the study variables, multilevel modeling was employed to analyze the direct effect of team-member exchange on team and individual job crafting, as well as the mediating effect of team job crafting between team-member exchange and individual job crafting.

Table 9 shows the relationships among team-member exchange, and team and individual job crafting. In Model 1, team-member exchange positively predicted individual job crafting (*β* = 0.49, *p* < 0.001); in Model 2, team-member exchange positively predicted team job crafting (*β* = 0.65, *p* < 0.001). This indicated that the higher the interaction quality of team members, the higher both the team and individual job crafting; thus, Hypothesis 1 and Hypothesis 2 were validated. In Model 3, team job crafting also positively predicted individual job crafting (*β* = 0.54, *p* < 0.001); Hypothesis 3 was supported. In Model 4, when both team-member exchange and team job crafting were entered for analysis, the *β* value decreased from 0.49 (*p* < 0.001) to 0.16 (*p* > 0.05). Team job crafting still positively predicted individual job crafting (*β* = 0.47, *p* < 0.001); the indirect relationship was 0.30 (*p* < 0.001). Team job crafting was fully mediating the relationship between team-member exchange and individual job crafting. Thus, team level team-member exchange could influence team job crafting and, in turn, influence individual job crafting. Hypothesis 4 was supported.

### 5.4. The Moderating Effect of Prevention Regulatory Focus

**Figure 2:**
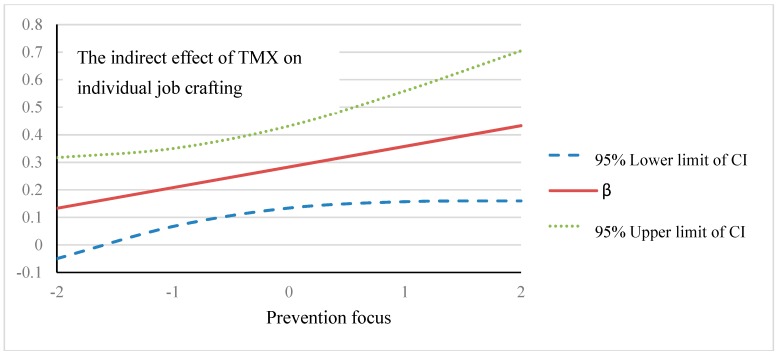
Moderating effect of prevention focus.

As shown in Table 10, team-member exchange relationships significantly predict job crafting (β = 0.646, *p* < 0.001). The interaction between job crafting and promotion regulatory focus was not significant (β = 0.047, *p* > 0.05) with 95% confidence interval values from −0.505 to 0.598. This indicates that promotion regulatory focus on the indirect relationship between team-member exchange and individual job crafting has no significant effect. Table 11 shows the prevention regulatory focus. Team-member exchange positively predicted team job crafting (β = 0.646, *p* < 0.001). The interaction of intergroup team-member exchange reached the marginal significance level (β = 0.378, *p* < 0.1); this indicates that the prevention regulatory focus still moderates the indirect relationship between team-member exchange and individual job crafting. The moderating effect of prevention regulatory focus is shown in Figure 2.

Figure 2 shows that, when individual prevention focus is low (−1.0 *SD*), the 95% confidence interval of the indirect effect fell to between 0.067 and 0.350. When individual prevention focus is high (+1.0 *SD*), the 95% confidence interval of indirect effect fell to between 0.157 and 0.559. When individual prevention regulatory focus is raised to 2.0 *SD* above the mean, the 95% confidence interval of indirect effect fell to between 0.160 and 0.705; i.e., 0 was still excluded. When individual prevention focus was −2.0 *SD* below the mean, the 95% confidence interval of indirect effect fell to between −0.050 and 0.317, indicating that, with the increase of prevention regulatory focus, the indirect effect of team-member exchange and individual job crafting will be higher.

### 5.5. Moderating Effects Differences Using Invariant Analysis

Invariant analysis was used to reveal the differences between individuals with prevention regulatory focus and promotion regulatory focus that would lead to a discrepancy in moderating effects on the indirect relationships of team-member exchange and individual job crafting.

Table 12 shows the results. The *β* value for high prevention regulatory focus was 0.663 (*p* < 0.05), and *SD* was 0.123; this study regarded these values as *β_1_* and *Seβ_1._* The *β* value for high promotion regulatory focus was 0.068 (*n.s*.), and *SD* was 0.177; this study regarded these values as *β_2_* and *Seβ_2_*. Both *β_1_* and *Seβ_1_* as well as *β_2_* and *Seβ_2._* were then applied to the following formula in equation (1). The discrepancy between high promotion regulatory focus and high prevention regulatory focus was 2.76, which exceeded the standard normal t distribution critical value (1.645). This implies that a difference exists between high promotion regulatory focus individuals and high prevention individuals. Regulatory focus moderated the indirect relationships between team-member regulatory focus and individual job crafting. Hypothesis 5 was supported.

(1) $$\frac{\left(\beta 1-\beta 2\right)}{\sqrt{{\left(Se\beta 1\right)}^{2}+{\left(Se\beta 2\right)}^{2}}}=\frac{\left(0.663-0.068\right)}{\sqrt{{\left(0.123\right)}^{2}+{\left(0.177\right)}^{2}}}=2.76$$

### 5.6. The Summarized Results

By means of rigorous statistical analysis, the final results of this study are summarized in Table 13.

## 6. Discussion

### 6.1. Academic Contribution

First, based on its findings, this study provides new insights about the factors that trigger individuals’ crafts and their jobs, and why we push forward our understanding. This strengthens the academic connection between individual and team level job crafting, which was not fully comprehended in previous studies.

Second, through a cross-level analysis, this study explains why relationships between team members affect individual job crafting behaviors. This study examines the influence of different types of regular focus, which have a different impact on individual job crafting, and explores how personal motivation affects individual job crafting behavior approaches. Based on the results of this study, with team members’ interaction quality increase, individuals’ job crafting frequencies would be higher. This finding echoed the study results of [5] and pushed forward our understanding of the way team level members’ interaction quality positively predicts individual job crafting frequency, thereby filling a gap inherent in exclusively individual-level studies. This study not only revealed the possible precursors of individual job crafting [52], but also expanded the discovery of possible antecedents of team job crafting via validating the connections of quality team members’ interaction and team job crafting [3].

Third, this study revealed the relationships between team-member exchange and individual job crafting. Furthermore, it found that team-member job crafting mediated the relationships between team-member exchange and individual job crafting.

Finally, this study is in response to the call for including TMX or team-member job crafting and individual factors, such as regulatory focus, to predict individual job crafting [3,13]. Individuals with a high prevention regulatory focus were more likely to enhance the indirect relationships of TMX on individual job crafting than those with a high promotion regulatory focus. This was because individuals with a high prevention focus tended to take more conservative measures than their colleagues to prevent resource losses, such as destroying interpersonal relationships, causing emotional exhaustion, and increasing work stress [16]. As to their decision-making processes, they were more likely to rely on team job crafting instead of individual job crafting. This behavior type was similar to Chinese cultural expectations that were in favor of collectivism to eliminate some unnecessary interpersonal problems.

### 6.2. Management Implications

Based on the above empirical analysis, this study provides suggestions to improve the current practice. First, this study argues that a good team-member exchange quality can trigger positive job crafting behavior. Therefore, team or organizational leaders should set up various opportunities to increase members’ interaction, such as opening discussions and knowledge sharing, thereby increasing the frequency of team job crafting behaviors and individual job crafting behaviors, which are beneficial for team leaders and their subordinates in eliciting more job crafting and working toward a common goal. When a common goal is achieved, it will likely increase the sense of mutual trust and work efficacy.

Second, this study showed that team job crafting promoted individual job crafting. Therefore, an organization could establish a positive organizational climate to increase opportunities for teamwork cooperation by setting common goals. When colleagues work together to collaborate on their common goals, team members will feel empowered to be responsible for their work and for meeting their role expectations granted by the team.

Nevertheless, social norms were very important when conducting team job crafting. According to social cognitive theory, various learning effects will apply to the team members as future action guidelines [53] (p. 86). Thus, at the emergent stage of team formation, it was important to establish a role model. In other words, each newcomer could also be assigned an experienced colleague as a mentor to coach them on everything about the team or organization, including actively showing how to conduct work tasks and acquire effective skills, thinking styles, and environmental management. Through establishing a mentoring program or system, the new staff member’s work behavior is expected by the company.

Third, effective management of individuals with a different regulatory focus is important. The organization should modify their management measures in accordance with their regulatory focus profiles. For example, prevention regulatory focus individuals are more likely tuning their job crafting in reference to environmental or other circumstantial cues. Conversely, if the environment influences are not salient, these prevention regulatory individuals may not perform as the organization expected. Thus, group leaders could borrow from positive group power concepts to clearly explain what the organizational expectations are and let them know that detrimental loss may eventuate if they fail to achieve organizational expectations. As to high promotion regulatory individuals, the supervisor should pay more attention to revealing whether or not their subordinates’ work styles meet the organization’s needs and expectations, or assign them tasks that do not interfere with other colleagues. Therefore, we can choose the right person to be a team member in the new team through measuring individual motivations when recruiting new staff or organizing new teams.

From the viewpoint of a member in an organization, prevention regulatory focus individuals should put more environmental elements into consideration, including team job crafting behavior frequency and organizational climates, to further examine whether these elements fit into individuals’ needs and are helpful in seeking resources to craft their jobs to meet individual personality traits, capacities, and levels of enthusiasm.

### 6.3. Limitations of this Study

This study’s limitations are mostly inherited from the methodology. First, self-report inventory reports are partially influenced by social expectations. For example, when team members were asked about their interpersonal relationships, they tended to give a more conservative reply when the answer was on the negative side to maintain their good impression within the team. We hid the participants’ demographic identification information and psychological constructs information for the inventory items to prevent such answers [54]. However, future studies can also add social expectation elements to the instruments to serve as a control variable. Second, owing to some sampling restrictions, the participant recruiting processes could not be fully random, so sampling error might not be eliminated. Third, this study used a two-stage data collection process; however, many factors may intervene to influence participants’ job crafting, which makes it challenging to establish causal conclusions of job crafting changes. Fourth, most instruments were derived from studies from other countries; although we did our best to prevent biases caused by cultural differences, some tiny problems may exist. These should not influence the study results as a whole, but we must still be cautious.

### 6.4. Suggestions for Future Studies

This research mainly explores the reasons and mechanisms that affect job crafting, but does not further explore the impact of job crafting on the performance of enterprises and organizations. This has affected the theoretical depth and explanatory power of this research to a certain extent. In fact, job crafting is a reaction to team-member dynamics, and some scholars have conducted in-depth research on team-member dynamics. For example, in [55], the impact of industry tournament incentives for CEO mobility and corporate performance is empirically analyzed. By means of classic agency literature, in [56] whether the number two executive in a firm could possibly mitigate the agency problems of the CEO was tested, and the authors found that the effect of mutual monitoring on firm performance is of greater significance and magnitude when other aspects of corporate governance are weak according to conventional measures. Meanwhile, in [57], the implications of mutual monitoring for firm performance and policy is clarified, and it is suggested that the effect of mutual monitoring on firm performance is more prominent in firms where the number two executive has sufficient incentive to monitor and where the information asymmetry between the board and the CEO is high. Therefore, future research can start from the perspective of team-member dynamics to further study the relationship between job crafting and enterprise, and team performance, in order to gain more theoretical knowledge.

According to the study results, it is possible for us to explore the causal relationships between team-member job crafting and individual job crafting. Previous literature had illustrated that team and individual job crafting were not mutually exclusive [5]. Thus, this study merely tried to explore how team job crafting influences individual job crafting; however, it is known that an individual’s behavior could be interactively influenced by personal, environmental, and behavioral factors. It is also possible for individual job crafting to affect team job crafting. Some dyad studies, such as [13], indicated that individual job crafting affected team job crafting; nevertheless, they did not probe the fluid type of job crafting within a team by means of team-based, cross-level viewpoints. More researchers could use qualitative or longitudinal inquiry methods and multi-stage, self-report inventories to reveal job crafting fluidity within a team in the future.

There are several job crafting viewpoints. This study used the four types of job crafting aspects [15], namely, increasing work resources, increasing interpersonal resources, increasing challenging work demands, and decreasing obstacle work demands. The current study regarded these four as a whole job crafting concept; accordingly, we did not differentiate their effects. Future studies could be done to reveal whether team circumstance differences lead to promoting or preventing a particular type of job crafting.

## 7. Conclusions

Some conclusions were obtained from the study. First, team-member exchange positively predicted individual job crafting and team job crafting, respectively. The higher the team-member exchange, the higher the team job crafting and individual job crafting. Second, team job crafting positively predicted individual job crafting. In other words, higher levels of team job crafting imply higher levels of individual job crafting. Third, team job crafting fully mediated the relationships between team-member exchange and individual job crafting. Fourth, prevention regulatory focus moderated the relationship between team-member exchange and individual job crafting. The invariant analysis indicated the relationships between team-member exchange and individual job crafting were different owing to individual personality traits. Individuals with a high prevention focus compared to individuals with a high promotion focus were more likely to increase team-member exchange to influence the relationships between team job crafting and individual job crafting.

## Figures and Tables

**Table 1:** Team-member exchange (TMX) scale.

	Items
1	I often suggest better work methods to others.
2	Others let me know when I affect their work.
3	I let others know when they affect my work.
4	Other members recognize my potential.
5	Other members understand my problem.
6	I often ask others for help.
7	I often volunteer extra help.
8	I am flexible about switching jobs with others.
9	I am willing to finish work assigned to others
10	Others are willing to finish work assigned to me.

α = 0.83.

**Table 2:** Positive individual job crafting.

	Items
**Increasing structural job resources**	
1	I try to develop my capabilities.
2	I try to develop myself professionally.
3	I try to learn new things at work.
4	I make sure that I use my capacities to the fullest.
5	I decide on my own how I do things.
**Increasing social job resources**	
6	I ask my supervisor to coach me.
7	I ask whether my supervisor is satisfied with my work.
8	I look to my supervisor for inspiration.
9	I ask others for feedback on my job performance.
10	I ask colleagues for advice.
**Increasing challenging job demands**	
11	When an interesting project comes along, I offer myself proactively as project co-worker.
12	If there are new developments, I am one of the first to learn about them and try them out.
13	When there is not much to do at work, I see it as a chance to start new projects.
14	I regularly take on extra tasks even though I do not receive extra salary for them.
15	I try to make my work more challenging by examining the underlying relationships between aspects of my job.

α = 0.89.

**Table 3:** Negative individual job crafting.

	Items
**Decreasing hindering job demands**	
1	I make sure that my work is mentally less intense.
2	I try to ensure that my work is emotionally less intense.
3	I manage my work so that I try to minimize contact with people whose problems affect me emotionally.
4	I organize my work so as to minimize contact with people whose expectations are unrealistic.
5	I organize my work in such a way to make sure that I do not have to concentrate for too long a period at once.
6	I make sure that my work is mentally less intense.

α = 0.72.

**Table 4:** Positive team job crafting.

	Items
**Increasing structural job resources**	
1	On the team, everyone tries to develop their capabilities.
2	On the team, everyone tries to develop their profession.
3	On the team, everyone tries to learn new things at work.
4	On the team, everyone makes sure that they use their capacities to the fullest.
5	On the team, we decide on their own how we do things.
**Increasing social job resources**	
6	On the team, we ask our supervisor to coach us.
7	On the team, everyone asks whether our supervisor is satisfied with their work.
8	On the team, we look to our supervisor for inspiration.
9	On the team, everyone asks others for feedback on individual job performance.
10	On the team, everyone asks colleagues for advice.
**Increasing challenging job demands**	
11	On the team, when an interesting project comes along, everyone offers themselves proactively as a project co-worker.
12	On the team, if there are new developments, everyone is one of the first to learn about them and try them out.
13	On the team, when there is not much to do at work, everyone sees it as a chance to start new projects.
14	On the team, everyone regularly takes on extra tasks even though they do not receive extra salary for them.
15	On the team, everyone tries to make their work more challenging by examining the underlying relationships between aspects of their job.

**Table 5:** Negative team job crafting.

	Items
**Decreasing hindering job demands**	
1	On the team, everyone makes sure that their work is mentally less intense.
2	On the team, everyone tries to ensure that their work is emotionally less intense.
3	On the team, everyone manages their work so that they try to minimize contact with people whose problems affect them emotionally.
4	On the team, everyone organizes their work so as to minimize contact with people whose expectations are unrealistic.
5	On the team, everyone tries to ensure that they do not have to make many difficult decisions at work.
6	On the team, everyone organizes their work in such a way to make sure that they do not have to concentrate for too long a period at once.

**Table 6:** Promotion focus.

	Items
1	I frequently imagine how I will achieve my hopes and aspirations.
2	I often think about the person I would ideally like to be in the future.
3	I typically focus on the success I hope to achieve in the future.
4	I often think about how I will achieve academic success.
5	My major goal in school right now is to achieve my academic ambitions.
6	I see myself as someone who is primarily striving to reach my “ideal self”—to fulfill my hopes, wishes, and aspirations.
7	In general, I am focused on achieving positive outcomes in my life.
8	I often imagine myself experiencing good things that I hope will happen to me.
9	Overall, I am more oriented toward achieving success than preventing failure.

α = 0.91.

**Table 7:** Prevention focus.

	Items
1	In general, I am focused on preventing negative events in my life.
2	I am anxious that I will fall short of my responsibilities and obligations.
3	I often think about the person I am afraid I might become in the future.
4	I often worry that I will fail to accomplish my academic goals.
5	I often imagine myself experiencing bad things that I fear might happen to me.
6	I frequently think about how I can prevent failures in my life.
7	I am more oriented toward preventing losses than I am toward achieving gains.
8	My major goal in school right now is to avoid becoming an academic failure.
9	I see myself as someone who is primarily striving to become the self I “ought” to be—to fulfill my duties, responsibilities, and obligations.

α = 0.85.

**Table 8:** The mean, SD, and Pearson’s correlation coefficients for study variables.

	M	SD	1	2	3	4
1. team-member exchange	3.84	0.24				
2. team job crafting behaviors	3.49	0.32	0.551 **			
3. promotion focus	4.00	0.47	0.220 **	0.284 **		
4. prevention focus	3.55	0.56	0.080	0.109 *	0.229 **	
5. individual job crafting	3.57	0.50	0.253 **	0.370 **	0.558 **	0.286 **

*N* = 364; * *p* < 0.05 ** *p* < 0.01.

**Table 9:** The relationships among team-member exchange behaviors, and individual and team job crafting behaviors.

Model	Individual Job Crafting	Team Job Crafting	Individual Job Crafting			
	Model 1	Model 2	Model 3	Model 4	95% CI	
**Within Level**					**Lower Limit**	**Upper Limit**
High school and below	−0.178 *		−0.124	−0.136	−0.301	0.029
Junior College	−0.054		−0.002	−0.009	−0.171	0.152
Undergraduate	−0.036		−0.039	−0.041	−0.147	0.064
Less than 1 year	0.161 *		0.123	0.127	−0.026	0.281
1–3 years	0.025		0.002	0.005	−0.114	0.124
3–5 years	0.015		0.002	0.001	−0.185	0.187
5–7 years	−0.004		−0.015	−0.014	−0.188	0.159
7–10 years	0.143		0.147	0.141	−0.049	0.332
*R* ^2^	0.036 *		0.024	0.026		
**Between Level**						
TMX	0.485 ***	0.646 ***		0.162	−0.106	0.431
Team job crafting			0.536 ***	0.468 ***	0.278	0.658
*R* ^2^	0.299 *	0.254 ***	0.562 ***	0.581 ***		
Indirect effect				0.302 ***	0.144	0.460
Total effect				0.464 ***	0.219	0.710

*n* = 364; * *p* < 0.05, *** *p* < 0.001.

**Table 10:** Moderated mediation of promotion regulatory focus.

Model	*β*	*(SE)*	95% CI		*R* ^2^
			Lower Limit	Upper Limit	
**Individual Job Crafting** **—Within Level**					
High school and below	−0.027	(0.08)	−0.191	0.137	0.298 ***
Junior College	0.020	(0.07)	−0.115	0.155	
Undergraduate	−0.019	(0.04)	−0.103	0.065	
Less than 1 year	0.121	(0.08)	−0.026	0.268	
1–3 years	0.022	(0.06)	−0.097	0.141	
3–5 years	−0.026	(0.08)	−0.180	0.128	
5–7 years	0.041	(0.08)	−0.115	0.197	
7–10 years	0.130	(0.09)	−0.041	0.301	
Promotion focus	0.496 ***	(0.05)	0.397	0.595	
**Team Job Crafting** **—Between Level**					
TMX	0.646 ***	(0.13)	0.384	0.907	0.254 **
**Individual job crafting**					
TMX	0.041	(0.12)	−0.197	0.280	0.384 *
Team job crafting	0.306 **	(0.11)	0.089	0.523	
Promotion focus	0.091	(0.11)	−0.118	0.300	
Team job crafting × promotion focus	0.047	(0.28)	−0.505	0.598	

*N* = 364; * *p* < 0.05, ** *p* < 0.01, *** *p* < 0.001.

**Table 11:** Moderated mediation of prevention regulatory focus.

Model	*β*	*(SE)*	95% CI		*R* ^2^
			Lower Limit	Upper Limit	
**Within Level**					
High school and below	−0.212 *	(0.08)	−0.375	−0.050	0.092 **
Junior College	−0.065	(0.09)	−0.233	0.102	
Undergraduate	−0.048	(0.05)	−0.148	0.052	
Less than 1 year	0.074	(0.08)	−0.076	0.225	
1–3 years	−0.045	(0.06)	−0.163	0.073	
3–5 years	−0.037	(0.09)	−0.216	0.142	
5–7 years	−0.047	(0.08)	−0.212	0.118	
7–10 years	0.072	(0.09)	−0.098	0.243	
Prevention focus	0.203 ***	(0.05)	0.105	0.301	
**Team job crafting Between Level**					
TMX	0.646 ***	(0.13)	0.384	0.907	0.254 **
**Individual job crafting**					
TMX	0.438 ***	(0.09)	−0.091	0.405	0.674 **
Team job crafting	0.157	(0.13)	0.261	0.616	
Prevention focus	0.098	(0.09)	−0.085	0.281	
Team job crafting × prevention focus	0.378 ^+^	(0.21)	−0.029	0.786	
**Indirect Effect**					
Prevention focus −2SD	0.133	(0.09)	−0.050	0.317	
Prevention focus −1SD	0.208 **	(0.07)	0.067	0.350	
Prevention focus MED	0.283 ***	(0.08)	0.134	0.432	
Prevention focus +1SD	0.358 ***	(0.10)	0.157	0.559	
Prevention focus +2SD	0.433 **	(0.14)	0.160	0.705	

*n* = 364; * *p* < 0.05, ** *p* < 0.01, *** *p* < 0.001, ^+^ 0.05 < *p* < 0.10.

**Table 13:** Summarization of results.

Research Hypothesis	Results
**Hypothesis 1.** Team-member exchange relationships can positively affect individual job crafting.	Supportive
**Hypothesis 2.** Team-member exchange relationships can positively affect team job crafting.	Supportive
**Hypothesis 3.** Team job crafting can positively affect individual job crafting.	Supportive
**Hypothesis 4.** Team-member exchange relationships, which positively affect individual job crafting, can regulate a moderated mediating effect through team modeling behavior.	Supportive
**Hypothesis 5.** Regulatory focus can moderate the indirect relationship between the team-member exchange relationships and individual job crafting. Those with a prevention focus can enhance the indirect relationship between team-member exchange relationships and individual job crafting. Those with a high-promotion focus are less likely to display a moderating effect.	Supportive

**Table 12:** High prevention and high promotion regulatory focus moderates the relationship between individual and team job crafting.

Group	High Prevention		High Promotion	
Model	Individual Job Crafting		Individual Job Crafting	
	*β*	*(SE)*	*β*	*(SE)*
*Between level*				
TMX	0.004	0.137	0.394 *	0.171
Team job crafting	0.663 ***	0.123	0.068	0.177
*R* ^2^	0.577 ***		0.529	

*n*_prevention focus_ = 186; *n*_promotion focus_ = 179; * *p* < 0.05, ** *p* < 0.01, *** *p*< 0.001.

## References

[1] Bakker A.B., Tims M., Derks D. (2012). Proactive personality and job performance: The role of job crafting and work engagement. Hum. Relat..

[2] Tims M., Bakker A.B., Derks D., Van Rhenen W. (2013). Job crafting at the team and individual level: Implications for work engagement and performance. Group Organ. Manag..

[3] Berg J.M., Dutton J.E., Wrzesniewski A., Dik B.J., Byrne Z.S., Steger M.F. (2013). Job crafting and meaningful work. Purpose and Meaning in the Workplace.

[4] Petrou P., Demerouti E., Peeters M.C.W., Schaufeli W.B., Hetland J. (2012). Crafting a job on a daily basis: Contextual correlates and the link to work engagement. J. Organ. Behav..

[5] Leana C., Appelbaum E., Shevchuk I. (2009). Work process and quality of care in early childhood education: The role of job crafting. Acad. Manag. J..

[6] McClelland G.P., Leach D.J., Clegg C.W., McGowan I. (2014). Collaborative crafting in call centre teams. J. Occup. Organ. Psychol..

[7] Vaskova R. (2007). Teamwork and High Performance Work Organization. https://www.eurofound.europa.eu/publications/article/2007/teamwork-and-high-performance-work-organisation.

[8] Wrzesniewski A., Dutton J.E. (2001). Crafting a job: Revisioning employees as active crafters of their work. Acad. Manag. Rev..

[9] Salas E., Shuffler M.L., Thayer A.L., Bedwell W.L., Lazzara E.H. (2015). Understanding and improving teamwork in organizations: A scientifically based practical guide. Hum. Resour. Manag..

[10] Hoegl M., Parboteeah K.P., Gemuenden H.G. (2003). When teamwork really matters: Task innovativeness as a moderator of the teamwork–performance relationship in software development projects. J. Eng. Technol. Manag..

[11] Dachner A.M., Miguel R. (2015). Job crafting: An unexplored benefit of friendships in project teams. SAM Adv. Manag. J..

[12] Farmer S.M., Van Dyne L., Kamdar D. (2015). The contextualized self: How team-member exchange leads to coworker identification and helping OCB. J. Appl. Psychol..

[13] Bakker A.B., Rodríguez-Muñoz A., Sanz Vergel A.I. (2016). Modelling job crafting behaviours: Implications for work engagement. Hum. Relat..

[14] Brass D.J., Burkhardt M.E. (1993). Potential power and power use: An investigation of structure and behavior. Acad. Manag. J..

[15] Tims M., Bakker A.B. (2010). Job crafting: Towards a new model of individual job redesign. S. Afr. J. Ind. Psychol..

[16] Higgins E.T., Shah J., Friedman R. (1997). Emotional responses to goal attainment: Strength of regulatory focus as moderator. J. Pers. Soc. Psychol..

[17] Wrzesniewski A., Berg J.M., Dutton J.E. (2010). Turn the job you have into the job you want. Harv. Bus. Rev.

[18] Seers A. (1989). Team-member exchange quality: A new construct for role-making research. Organ. Behav. Hum. Dec. Process..

[19] Seers A., Petty M.M., Cashman J.F. (1995). Team member exchange under team and traditional management: A naturally occurring quasi-experiment. Group Organ. Manag..

[20] Liao H., Liu D., Loi R. (2010). Looking at both sides of the social exchange coin: A social cognitive perspective on the joint effects Of relationship quality and differentiation On creativity. Acad. Manag. J..

[21] Bandura A. (1986). Social Foundations of Thought and Action.

[22] Homans G.C. (1958). Social behavior as exchange. Am. J. Sociol..

[23] Weick K.E. (1979). The Social Psychology of Organizing.

[24] Brewer M.B., Gardner W. (1996). Who is this “We”? Levels of collective identity and self representations. J. Pers. Soc. Psychol..

[25] Bandura A. (2000). Exercise of human agency through collective efficacy. Curr. Dir. Psychol. Sci..

[26] Ghitulescu B. (2006). Job Crafting and Social Embeddedness at Work. Ph.D. Thesis.

[27] Torrente P., Salanova M., Llorens S., Schaufeli W.B. (2012). Teams make it work: How team work engagement mediates between social resources and performance On the teams. Psicothema.

[28] Cannon-Bowers J.A., Salas E. (2001). Reflections on shared cognition. J. Organ. Behav..

[29] Taggar S., Ellis R. (2007). The role of leaders in shaping formal team norms. Leadersh. Q..

[30] Abelson R.P., Carroll J.S., Payne W.J. (1976). Script processing in attitude formation and decision making. Cognition and Social Behavior.

[31] Barker J.R. (1993). Tightening the iron cage: Concertive control in self-managing teams. Adm. Sci. Q..

[32] Postmes T., Spears R., Lea M. (2000). The formation of group norms in computer-mediated communication. Hum. Commun. Res..

[33] Caldwell D.F., O’Reilly C.A. (2003). The determinants of team-based innovation in organizations: The role of social influence. Small Group Res..

[34] Edmondson A. (1999). Psychological safety and learning behavior in work teams. Adm. Sci. Q..

[35] Bandura A. (2001). Social cognitive theory: An agentic perspective. Annu. Rev. Psychol..

[36] Baron R.M., Kenny D.A. (1986). The moderator-mediator variable distinction in social psychological research: Conceptual, strategic, and statistical considerations. J. Pers. Soc. Psychol..

[37] Edwards J.R., Lambert L.S. (2007). Methods for Integrating Moderation and Mediation: A General Analytical Framework Using Moderated Path Analysis. Psychol. Methods.

[38] Muller D., Judd C.M., Yzerbyt V.Y. (2005). When Moderation is Mediated and Mediation is Moderated. J. Pers. Soc. Psychol..

[39] Tims M., Bakker A.B., Derks D. (2012). Development and validation of the job crafting scale. J. Vocat. Behav..

[40] Brockner J., Higgins E.T., Low M.B. (2004). Regulatory focus theory and the entrepreneurial process. J. Bus. Ventur..

[41] Crowe E., Higgins E.T. (1997). Regulatory focus and strategic inclinations: Promotion and prevention in decision-making. Organ. Behav. Hum. Dec. Process..

[42] Liberman N., Idson L.C., Higgins E.T. (2005). Predicting the intensity of losses vs. non-gains and non-losses vs. gains in judging fairness and value: A test of the loss aversion explanation. J. Exp. Soc. Psychol..

[43] Levine J.M., Higgins E.T., Choi H.-S. (2000). Development of strategic norms in groups. Organ. Behav. Hum. Dec. Process..

[44] Hsu H.H., Huang H.Y., Lin C.C., Peng T.K. (2015). Are You Exhausted, Good Soldiers? Exploring the Impacts of Extra-role Behaviors and Regulatory Focus on Emotional Exhaustion form a Resource Conservation Perspective. Organ. Manag..

[45] Keller P.A. (2006). Regulatory focus and efficacy of health messages. J. Consum. Res..

[46] Yang X.D. (2012). The Relationship between Social Support at Work and Job Crafting Behavior: Organization-Based Self-Esteem as Mediation and Achievement Motivation as Moderation. Master’s Thesis.

[47] Lockwood P., Jordan C.H., Kunda Z. (2002). Motivation by positive or negative role models: Regulatory focus determines who will best inspire us. J. Pers. Soc. Psychol..

[48] DeVellis R.F. (1991). Scale Development: Theory and Applications.

[49] Niessen C., Weseler D., Kostova P. (2016). When and why do individuals craft their jobs? The role of individual motivation and work characteristics for job crafting. Hum. Relat..

[50] Zacher H., Hacker W., Frese M. (2016). Action regulation across the adult lifespan (ARAL): A meta-theory of work and aging. Work Aging Retire..

[51] Podsakoff P.M., MacKenzie S.B., Lee J.-Y., Podsakoff N.P. (2003). Common method biases in behavioral research: A critical review of the literature and recommended remedies. J. Appl. Physiol..

[52] Wrzesniewski A., LoBuglio N., Dutton J.E., Berg J.M., Bakker A.B. (2013). Job crafting and cultivating positive meaning and identity in work. Advances in Positive Organizational Psychology.

[53] Bandura A. (1997). Self-Efficacy: The Exercise of Control.

[54] Butts M.M., Vandenberg R.J., Williams L.J. (2006). Investigating the susceptibility of measurement invariance tests: The effects of common method variance. Academy of Management Proceedings.

[55] Coles J.L., Li Z.C.F., Wang Y.A. (2018). Industry tournament incentives. Rev. Financ. Stud..

[56] Li Z.C.F. (2014). Mutual monitoring and agency problems. SSRN Electron. J..

[57] Li Z.C.F. (2014). Mutual monitoring and corporate governance. J. Bank. Financ..

